# The Ethics of Medical Visiting

**Published:** 1888-12

**Authors:** 


### *THE ETHICS OF MEDICAL VISITING.*

The publication, in a recent number of the *Western Medical Reporter*, of a lengthy correspondence between Mr. Lawson Tait, and Dr. D. A. K. Steele, of Chicago, who had been denied admission to the former's operating room, suggests the questions, "What are the ethics of medical visiting?" What are the privileges to be accorded a visitor; what may a surgeon refuse, and still be within the elastic pale of "ethics?" What are the privileges of "natural selection" to be accorded a much used, and, perhaps, equally much abused surgeon like Mr. Tait? The ordinary surgeon generally finds it no inconvenience to admit to his clinic persons recommended, if due notice is given of their coming. Abdominal operations are, however, out of the ordinary, just as Mr. Tait, by reason of his position and prominence, is out of the ordinary. The attention he has received, favorable and unfavorable, from those who have chosen to visit and criticize him, is also out of the ordinary. The attention he has received in the present instance is also out of the ordinary. Let us examine it, impersonally.

Finding from his experience that he does not receive from visitors the courtesy due to him, Mr. Tait determines not to receive casual visitors. A firm stand upon this resolve seems, in the present instance, to be the head and front of his offending. Of his intention to make no exception to his rule in the case of Dr. Steele, he took pains to write to Dr. Jackson, who wrote introducing him. Here, certainly, was no discourtesy. The only question now involved is, was the act of refusal a discourtesy? So far as popular prejudice is concerned, which need be thrown out of account—so far as Mr. Tait is affected—his popularity among those who are the self-constituted critics of him and his methods, will be still less than it is. So far as the merits of the case are concerned with those who have seen, and are just enough to appreciate his work, they will wonder now more than ever, that he was so tardy in protecting himself from observers, who, whatever diligence they possess, too often have a "zeal, but not according to knowledge."

Says the complainant in the present instance: "Now, sir, I cannot but construe this as an act of discourtesy, * * * an insult to the medical profession of Chicago, * * * and to my colleagues in the 'College of Physicians and Surgeons.'"

In the same number of the *Western Medical Reporter* appears a letter from Dr. Steele, in which he recounts his experiences and impressions of other operators whom he has visited and observed. These are some of the impressions. Of Mr. Treves he says: "He is rather a careless antiseptician." Of Mr. Christopher Heath: "Rather given to profanity if matters don't go to suit him." Still another: "I saw Mr. Knowsley Thornton operate upon a unique case in rather a bungling manner." Yet another: "At my final visit, I saw a most interesting case of hemorrhagic cyst of the pancreas. Stephen McKenzie regarded it as an abdominal lipoma. Treves thought it to be a sarcoma, and I made bold to suggest to him in the ward * * * the strong probability of its being a pancreatic cyst. * * * To my delight, it proved to be, as I predicted, a cyst of the pancreas. At his next clinical lecture, Treves gave a very complete review of the literature of pancreatic cysts, omitting to mention, however, the recent American cases of Bull, Fenger, and my own. * * * He was of the impression that a correct diagnosis had never been made prior to the operation. I took occasion to supply the omissions and correct his misapprehensions on the subject in a personal letter."

At the risk of being tedious, we have quoted to this extent, simply for the intelligent criticism of our readers. Is it courteous for a visitor, courteously admitted to any operation, or society, by "the unwritten law of medical comity," [Steele to Tait] to go out from that operation, or that society, or interview, to characterize one, as careless in antiseptics; another, as profane; and still another, as a bungler, in any respect whatsoever? Is it a part of the unwritten law of medical comity to make a formal pronunciamento that Mr. Treves and Mr. McKenzie failed in their diagnosis of an abdominal growth, while Mr. Steele, of Chicago, correctly diagnosticated it? The modesty of such announcement goes without question. Leaving, for the moment, Mr. Tait's refusal out of sight, it would seem that such criticism as the above would justify any man's exclusion from a future operation, "by the unwritten law of medical comity."

We do not believe it generally felt that an operating-room is, or should be, a resort either of a scientific multitude, or of a curious rabble, or of eager critics. On the other hand, a surgeon has the right of every other man to protect both his work and his patients. The limits of that protection he must decide for himself. It is his right pure and simple, without question. No man disappointed in a failure to see an operation, has a right to interpret a polite refusal as a personal insult, or as a general slight to all his friends and associates.

The tone of Dr. Steele's letters is unfortunate for his side of the question. When he writes, "Why I Did not See the Birmingham Spayer," he shows, we think, the spirit in which he was ready to criticize Mr. Tait, and justifies a standpoint of still greater exclusiveness on his part, so far as admission into his operating-room is concerned. The "unwritten law of medical comity," like mercy, should "bless him that gives and him that takes," and should be always ready to allow to others what it exacts for itself. So far as this controversy will influence Mr. Tait and other surgeons—British or continental—in their opinion, and estimate of America's medical men, it is unfortunate. We trust, however, they will not follow the principle of *ex uno disce omnes,* but rather look on the "comity" side of the question and remember

> "'Tis pleasant sure to see one's name in print,
> A book's a book, although there's nothing in't."

Dr. D. B. St. John Roosa, of New York, in his anniversary address before the New York Academy of Medicine, delivered Thursday evening, November 15, 1888, in the presence of laity and profession, presented the question of divorcing the teaching and licensing power with reference to the practice of medicine in this State, in a most trenchant, convincing, and happy manner It was an argument, well-nigh unanswerable, in favor of creating a State Board of Examiners that shall have sole power to grant practising licenses to legally graduated applicants, a measure which the Erie County Medical Society originally presented to the Medical Society of the State of New York, and which the latter made several attempts to procure statutory enactment upon by the Legislature.

Dr. Roosa possesses the happy faculty of always stating his points in succinct, well-chosen—nay, even eloquent—sentences, and the last paragraph of his discourse is herewith presented (*New York Medical Journal*, November 24, 1888,) as a delightful specimen of metaphorical rhetoric:

"In one of the first months of the War of the Great Rebellion, a squadron of United States cavalry was galloping at night through the lanes of Northern Virginia. A young medical officer rode beside the captain in command. Suddenly, as evidence of a hostile force in ambush came to the trained eye or ear of the officer in command, a man whose experience had been long among the Indians of the frontier, there was the order, 'Halt!' After some hurried direction to the orderly sergeant, he concluded: 'Put the doctor in the center; we may need him.' So I ask you, while the physician shares with you the perils and vicissitudes of life, to care for him and his class, put them in the centre, safe from needless injury and unwarranted peril, and where they may, to their capacity, and in the proper time, help to maintain and preserve the Commonwealth."

---

A Physicians' Protective Alliance has recently been organized in Brooklyn, whose purpose is to protect the members from being defrauded of their fees by unworthy persons. The

Alliance accepts as members all legally qualified practitioners of medicine who may apply, upon payment of two dollars initiation fee and two dollars annual dues.

Each member will be entitled to a list of delinquents, which will be revised from time to time; but the objects of the Alliance are not to interfere with charitable service to the worthy poor. The *Brooklyn Medical Journal*, from which we condense the above, further says that "the present is too early in the history of the Alliance to judge accurately of its practical workings and final success. The meeting for organization was large and enthusiastic; the officers chosen include physicians from both schools of practice of the most conservative character and highest repute; the constitution adopted is comprehensive, yet guarded in all its features, and the interests of the debtor as well as the creditor have been jealously guarded. The wide-spread interest in the plan and purposes of the organization among the practitioners at large would seem to indicate that it is destined to play an important part in the future of medical practice in this city."

Perhaps the time is near at hand for a similar organization to be put into practical operation in Buffalo. Certainly there is an ample field for one to operate in, and with benefit to professional *espirit du corps*.

---

The American Association of Obstetricians and Gynecologists, which was organized in this city in April, 1888, held its first annual meeting in Washington, September 18, 19, and 20, 1888. From the character and standing of the men that go to make up its Fellowship, and from the nature and quality of the scientific work done by the Association at its first meeting, as evidenced by the abstract of its proceedings published in the *American Journal of Obstetrics* for October, it must be confessed that it compares favorably with the older societies that compose the Congress of American Physicians and Surgeons.

The Association seems to be made up of middle-aged and younger men, most of whom have already made something of a national reputation in their specialties, and all of whom are progressive, excellent exponents of modern obstetrical and gynecological medicine.

We quote the following from the *Medical Register*, Philadelphia, issue of October 27th, as a fair reflex of the opinion and judgment forming by the profession and journals upon this Association: "Nearly all the members have made many learned contributions to the medical press, and are well known in their specialties in this country and in Europe. The first meeting was held in Washington at the meeting of the Congress of Physicians and Surgeons; and the abstracts of the transactions published in the medical journals indicate that the complete proceedings, when given to the medical profession, will be of a high standard of scientific excellence."

---

We have received the circular of Prof. John A. Miller, Ph. D., who announces his laboratory and office at the Medical Department of Niagara University, and also that he is prepared to undertake all kinds of chemical analyses, as in cases of poisoning, of medicines and urine, as well as of industrial products. In announcing again Dr. Miller's work as a thorough and well-equipped chemist, we congratulate the profession that it has so accomplished a scientist to whom to refer all cases requiring the services of a skilled chemist. He possesses in his laboratory every convenience for his special work and also the training and education to use them. This addition to the corps of scientific workers is a great acquisition to the city and to the college with which he is connected. We bespeak for Dr. Miller the encouragement of an appreciative profession, and the success which comes from earnest and conscientious labor.

